# Effect of Chinese-Yam-Based Emulsion Gel on Beef Emulsification Characteristics

**DOI:** 10.3390/foods14040692

**Published:** 2025-02-17

**Authors:** Hao Zhang, Xujin Yang, Aiwu Gao, Limin Li

**Affiliations:** 1 College of Food Science and Engineering, Inner Mongolia Agricultural University, Hohhot 010018, China; zhanghao8530@163.com (H.Z.); eter88@163.com (X.Y.); 13848441713@163.com (L.L.); 2 College of Food Science and Engineering, Shandong Agricultural and Engineering University, Jinan 250100, China; 3 Inner Mongolia Agricultural University Yang Xujin Innovation Studio, Hohhot 010018, China

**Keywords:** fat substitute, emulsion gel, minced meat system, microstructural characteristics

## Abstract

Addressing the prevalent issue of excessive fat consumption in contemporary diets, a novel method has been devised for the preparation of an emulsion gel enriched with healthful fatty acids which possesses superior emulsification characteristics. This innovative approach aims to serve as a viable substitute for the fat content in beef mince. This paper aims to evaluate the effect of emulsion gels, formulated primarily with Chinese yam as the key ingredient, on the emulsification properties and microstructural characteristics of beef mince. The findings indicate that, when the proportion of fat substituted by the emulsion gel reaches 50%, the surface hydrophobicity of the beef mince attains a value of 37.34%, which approximates that of beef tallow. Furthermore, the water retention capacity of this formulation is significantly superior to that of the other test groups (*p* < 0.05). Additionally, when the fat replacement with emulsion gel is increased to 75%, the protein solubility is observed to be 22.85 mg/mL. As the quantity of emulsion gel increases, the gel structure of the beef mince undergoes a gradual densification. This alteration is accompanied by a statistically significant upward trend in the overall α-helix content (*p* < 0.05), whereas the β-turn angle exhibits an opposite trend and the β-sheet content demonstrates a biphasic pattern, initially increasing and subsequently decreasing. Notably, the random coil of the emulsion gel reaches its minimum when the fat content is at 50%, corresponding to a state of maximal stability in the beef mince. This study provides a basis for developing research into meat products with a healthier fat profile.

## 1. Introduction

As people’s diets gradually shift towards high-calorie diets, a series of diseases related to excessive fat intake have emerged, as well as the impact of the meat industry on the environment [1,2,3,4]. Research indicates that a mere reduction in the fat content of the formula can significantly impair the emulsifying properties of the product, which in turn directly influences its taste, texture, and overall stability [5]. Meat products with a healthier fat profile have become the current research focus.

Currently, the academic focus domestically and internationally on the matter of reducing fat content in meat products primarily revolves around strategies for decreasing or substituting the fat content within the formulation [6,7]. Scientific researchers have gathered substantial evidence that associates the intake of high-fat products with various health consequences. The removal of fat from such products, however, presents a formidable challenge, as fat not only contributes to the nutritional profile but also significantly influences the rheological and sensory attributes of the final product [8]. Traditional fat substitutes, which encompass cereal flour-based alternatives, protein-derived replacements, and pre-emulsification systems, fall short of adequately addressing the demand for mimicking the solid-like structural adipose tissue [9].

The utilization of solid-structured fats as substitutes for animal fats is employed to decrease the fat content and enhance the fatty acid composition in meat products, such as patties and sausages. Among these, oleogels [10] and emulsion gel [11] are the most prevalent options. Oil gels are distinguished by their low content of saturated and trans fatty acids. Furthermore, due to their excellent plasticity, they can serve as an ideal fat substitute without compromising the flavor profile of the food [12,13]. However, most of the gelling agents for preparing oil gels are not applicable to food, which restricts their application in low–fat meat products. When used as a fat substitute, emulsion gel shows an excellent fat reducing effect and oxidation stability, and is easy to prepare [14]. However, the research on emulsion gel mostly focuses on improving the fatty acid composition of the product, and there are few reports on its emulsification stability. The emulsifying characteristics of Chinese yam enable the production of a white gel that possesses a visual appearance analogous to fat. Furthermore, Chinese yam is abundant in polysaccharides and phenolic compounds, possessing notable antioxidant properties [15,16,17], which have the capacity to ameliorate the susceptibility to oxidation of the unsaturated fatty acids. Chinese yam powder contains a large amount of dietary fiber, which can promote gastrointestinal digestion and absorption, and it can be used as raw material to prepare emulsion gel.

The present experiment is designed to delve into the impact of emulsion gels formulated primarily from Chinese yam on the emulsifying characteristics and microstructural attributes of beef mince. This paper aims to evaluate the effect of emulsion gels, formulated primarily with Chinese yam as the key ingredient, on the emulsification properties and microstructural characteristics of beef mince. The study employs a suite of sophisticated evaluation metrics, including surface hydrophobicity, emulsion stability, water-holding capacity, protein solubility, protein secondary structure, intermolecular forces, and microstructural analysis. The ultimate goal is to identify a healthier fat substitute that exhibits enhanced emulsifying properties. This endeavor contributes novel methodologies and theoretical insights to the ongoing research efforts dedicated to exploring and developing innovative fat substitutes.

## 2. Materials and Methods

### 2.1. Materials

We used fresh, chilled lean beef from the hind legs of cattle, purchased from Hohhot Hualian Supermarket. The lean meat and fat were separated, and the fat was vacuum-packaged and stored in a −20 °C refrigerator. The sinews in the lean meat were removed, and then it was minced, ground, and weighed, and stored in a −20 °C refrigerator. One day before processing, one bag of homogenized raw meat was thawed each time during preparation. Chinese yam powder was purchased from Shanxi Angxu Biotechnology Co., Ltd., Xi’an, China. Cold-pressed flaxseed oil was purchased from Hohhot Hualian Supermarket. Soy protein isolate was provided by Soleibao Biotechnology Co., Ltd., Beijing, China. Calcium sulfate and sodium pyrophosphate were purchased from Macklin Biochemical Technology Co., Ltd., Shanghai, China; sodium alginate, bromophenol blue, dipotassium hydrogen phosphate, potassium dihydrogen phosphate, β mercaptoethanol, urea, glutaraldehyde, ethanol, and sodium chloride were purchased from Sinopharm Chemical Reagent Co., Ltd., Shanghai, China. All of the above reagents were of analytical purity.

### 2.2. Preparation of Emulsion Gels

The oil-in-water emulsion gel was formulated from white yam powder, cold-pressed flaxseed oil, water, and gelling agent. The gelling agent was composed of 0.73% sodium alginate, 0.73% CaSO_4_, and 0.54% sodium pyrophosphate. Referring to the method of Pintado for preparing emulsion gel [18], 3.76 g of soy protein isolate was first weighed and placed in a beaker, then 64 mL of distilled water was added and stirred with a magnetic stirrer (1500 rpm, 2 h) (HS 4, IKA, Staufen, Germany), and then left overnight. Before the experiment, it was first heated in a water bath (70 °C, 30 min) (HH2, Shanghai Fu Ma Test Equipment Co., Ltd., Shanghai, China), then cooled to about 50 °C, then had 20 g of cold-pressed flaxseed oil added to it. The content of cold-pressed flaxseed oil was determined as a fixed value, and stirred until evenly mixed with a high-speed disperser (7000 rpm, 10 min) (XHFDY, Ningbo Xin zhi Biotechnology Co., Ltd., Ningbo, China). We added 10 g of Chinese yam powder and gelling agent at the same time, and stirred with a mixer at 50 °C (360 rpm, 30 min) until evenly mixed. Finally, the sample was placed in a metal container under pressure to compact and prevent bubbles and was left for 24 h at 4 °C (BBCD290WX, Qingdao Haier Company, Qingdao, China) to obtain the emulsion gel sample.

### 2.3. Preparation of Minced Beef

The preparation of minced beef was also carried out according to the methods described in relevant references. We defrosted the beef before preparation (about 20 h at 4 °C) and stirred all of the raw materials into the meat grinder according to the recipe in Table 1. The evenly stirred mixture was prepared into balls (3.5 cm in diameter) and refrigerated at 4 °C for later use. Before the experiment, the sample taken from the refrigerator was left to stand at room temperature for 30 min. Each group of tests was repeated five times, and the average value was the final test value.

### 2.4. Determination of Surface Hydrophobicity

Samples of the 0.6 g raw mince were randomly weighed and suspended in 20 mL of 20 mmoL/L phosphate buffered (pH = 6.0) solution. The high-speed disperser was used to homogenize the meat for a period of time (10,000 rpm, homogenized 3 times, each time for 20 s) so that the mince was evenly distributed in the solution. A total of 1 mL of the homogenized solution was randomly removed and 100 μL of bromophenol blue was added. One hundred μL 1 mg/mL of bromophenol blue was added into 1 mL of phosphate buffer solution as control. Each group of samples was violently shaken for 20 min. Each set of samples was centrifuged using a centrifuge (6000× *g*, 4 °C, 15 min) (ALLEGRA X-30R (B06322), Beckman Coulter Ltd., Brea, CA, USA). After centrifugation, 0.5 mL of the supernatant (the clear liquid above the sediment in the centrifuge tube) was pipetted from both the test group and control group samples. To this, 4.5 mL of phosphate buffer solution was added. The absorbance value of each mixture was then determined at 595 nm using a spectrophotometer (U-2910, Hitachi corporation, Tokyo, Japan). Each treatment group was measured in parallel five times [19], and Formula (1) is as follows:

(1) $$\mathrm{Bound}\;bromophenol\;blue/\mu g=100\;\mu g\times \frac{A_{contrast}-A_{sample}}{A_{contrast}}$$

### 2.5. Determination of Emulsion Stability

The emulsification stability of beef mince refers to the ability of the stable emulsion formed, comprising fat and water within the beef mince, to maintain its homogeneity without layer separation or precipitation during the emulsification process. The specific determination method is as follows:

The beef mince was randomly weighed (*M*_1_) and placed in a 50 mL centrifuge tube (*M*_0_) with screw cap, centrifuged (150× *g*, 3 min), then sealed and placed in a constant temperature water bath at 70 °C for 30 min. The centrifuge tube was removed and the screw cap removed, and placed upside down on a weighted petri dish (*M*_2_) for 1 h, and then we weighed the centrifuge tube and determined the total weight of mince (*M*_3_), heated the juice collected from the petri dish at 103 °C for 16 h (DGX-9143BC, Shanghai Fu Ma Test Equipment Co., Ltd., Shanghai, China), and finally measured the total weight after heating (*M*_4_). Each treatment group was measured in parallel five times, and Formulas (2)–(5) are as follows:

(2) $$Total\;juice\;loss/\%=(M_{0}+M_{1})-M_{3}$$

(3) $$Percentage\;of\;total\;juice\;loss/\%=\frac{TEF}{M_{1}}\times 100$$

(4) $$Percentage\;of\;water\;loss/\%=\frac{\left(M_{1}+M_{0}-M_{3})-(M_{4}-M_{2}\right)}{M_{1}}\times 100$$

(5) $$Fat\;loss\;percentage/\%=\frac{M_{4}-M_{2}}{TEF}\times 100$$

### 2.6. Determination of Water Retention

Each group randomly weighed about 5 g of raw mince into a 10 mL centrifuge tube that had been weighed in advance, and then put it in a water bath at 85 °C for 15 min until the central temperature reached 75 °C. The sample was weighed after moisture was absorbed by filter paper. Each treatment group was measured in parallel five times [20], and Formula (6) is as follows:

(6) $$Cooking\;loss(\%)=\frac{W_{1}-W_{2}}{W}\times 100\%$$

where *W*_1_ and *W*_2_, respectively, represent the weight of minced meat before and after cooking, in grams. *W* represents the weight of meat, in grams.

Each group randomly weighed about 5 g of raw mince into a 10 mL centrifuge tube that had been weighed in advance, then weighed the sample and centrifuged it (17,000× *g*, 10 min, 4 °C), and then the centrifuged water was sucked out with filter paper and the centrifuged sample was weighed again. Each treatment group was measured in parallel five times, and Formula (7) is as follows:

(7) $$WHC=\frac{M_{2}-M_{0}}{M_{1}-M_{0}}\times 100\%$$

where *M*_1_ and *M*_2_ represent the mass of minced meat before and after centrifugation, respectively, in grams; *M*_0_ represents the mass of the centrifuge tube, in grams.

### 2.7. Determination of Protein Solubility

We referred to Xu’s method of measuring total protein solubility [21] and made some modifications on this basis. A frozen muscle sample (1 g) was homogenized in an ice bath of 20 mL of cold 0.1 moL/L potassium phosphate buffer (pH = 7.2, 1.1 moL/L KI) or 10 mL of 0.025 moL/L potassium phosphate buffer (pH = 7.2) to determine total protein solubility and sarcoplasmic protein solubility, respectively. Then, the homogenate was centrifuged at 4 °C at 5000× *g* for 20 min, and the protein concentration in the supernatant was determined by the Biuret method [22]. The solubility of myofibrillar protein was calculated by subtracting the solubility of sarcoplasmic protein from the total protein solubility.

### 2.8. Determination of Protein Secondary Structure

The amide I band (1600–1700 cm^−1^) was commonly used to analyze the changes in the protein secondary structure. The wave number range of 1610–1639 cm^−1^ was usually considered as β-sheet, 1640–1649 cm^−1^ as random coil, 1650–1659 cm^−1^ as α-helix, and 1660–1700 cm^−1^ as β-turn [23].

The protein secondary structure of minced beef samples was determined by Near Infrared Spectrum Instrument (PerkinElmer Frontier, Perkinelmer Enterprise Management (Shanghai) Co., Ltd., Shanghai, China). Samples of minced beef were frozen at −20 °C for about 4 h, after which the samples were freeze-dried in a vacuum freeze-dryer (Foring, Sihuan Freke Technology Development (Beijing) Co., Ltd., Beijing, China) until they were completely dry. The fully dried samples were pounded into powder and stored in a dryer until used. The range included a 4000–400 cm^−1^ region at 4 cm^−1^ resolution (room temperature), and single-beam mode intervals were used for 32 scans. Peak-Fit 4.12 software was used for curve fitting, and the secondary structure content of minced meat was calculated through curve fitting to the reverse second derivative amide I band of 1600–1700 cm^−1^ [24,25].

### 2.9. Determination of Intermolecular Forces

We accurately weighed 3 g minced beef samples which we evenly dispersed into 15 mL samples of five solvents: 0.05 moL/L Sodium chloride (S1), 0.6 moL/L sodium chloride (S2), 0.6 moL/L sodium chloride + 1.5 moL/L urea (S3), 0.6 moL/L sodium chloride + 8 moL/L urea (S4), and 0.6 moL/L sodium chloride + 8 moL/L urea + 1.5 moL/L β- mercaptoethanol (S5). The samples were homogenized (10,000 rpm, 1 min) with a high-speed disperser, and after homogenization, the samples were left for 1 h at 4 °C and then centrifuged (10,000× *g*, 10 min) to obtain the supernatant. The protein content was determined by Lowry method. The ionic bond content was expressed as the difference between the protein content dissolved in S2 and S1. The hydrogen bond content was expressed as the difference between the protein content dissolved in S3 and S2. The number of hydrophobic interactions was expressed as the difference between the amount of protein dissolved in S4 and S3. The disulfide bond content was expressed as the difference between the protein content dissolved in S5 and S4. The result was the average of the three repetitions and was expressed as the proportion of each grade (expressed as % of total protein) [26,27].

### 2.10. Determination of Microstructure

The cooked minced beef samples were cut into circular slices with double-sided blades, fixed with glutaraldehyde for 3 h, and then cleaned. The cleaned samples were dehydrated with ethanol (50%, 70%, 90%, 95%, and 100%) in gradient, with each concentration treated for 30 min. Then, the samples of each group were freeze-dried, and finally, the microstructure was observed using scanning electron microscopy (SEM) (TM4000Pul SEM: Hitachi Manufacturing Co., Ltd., Chiyoda, Japan). The acceleration voltage of SEM was 15 kV, and the samples were measured in parallel 5 times [28].

### 2.11. Statistics

Each group of tests was repeated five times, and the average value was the final test value. The data were presented as mean ± standard deviation. The data were processed and analyzed using IBM SPSS 26.0 software. One-way analysis of variance (Duncan test) was used to examine the significance of the differences in the results of each group. *p* < 0.05 indicates a significant difference. Graphs were plotted by Origin 2024 64Bit software.

## 3. Results and Discussion

### 3.1. Emulsification Characteristics

#### 3.1.1. Effect of Chinese-Yam-Based Emulsion Gel on the Surface Hydrophobicity of Minced Beef

**Figure 1:**
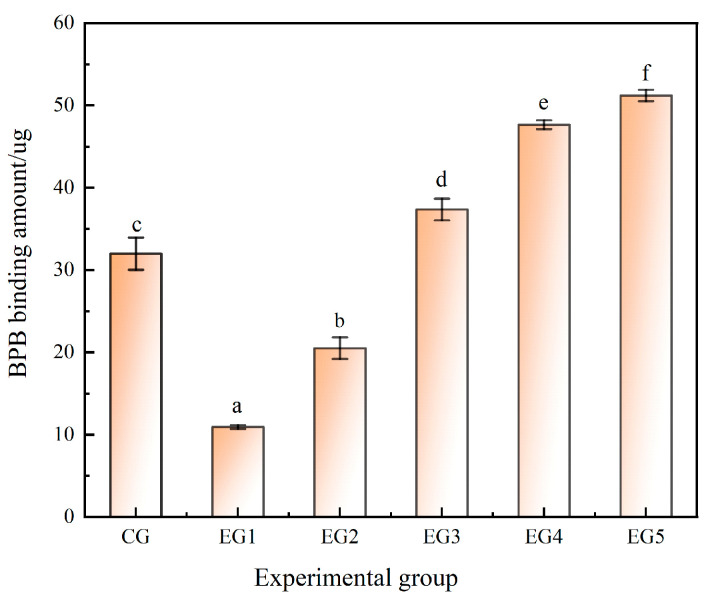
Surface hydrophobicity of beef minced with addition of different amounts of emulsion gel. Note: Amount of bound bromophenol blue is difference between total bromophenol blue and free bromophenol blue. Different letters indicate that there are significant differences between experimental groups (*p* < 0.05).

BPB is bromophenol blue, which is a pH indicator, and its ability to bind to proteins can reflect the surface hydrophobicity of proteins. Surface hydrophobicity can reflect the distribution of hydrophobic amino acids on the surface of proteins, which is closely related to the functional properties of proteins. Before the experiment, the beef mince was removed from the refrigerator and kept at room temperature for 30 min until it completely softened. As shown in Figure 1, the surface hydrophobicity of the emulsion gel fat substitute gradually increased with the increase in the proportion of emulsion gel; there were significant differences between the groups (*p* < 0.05). The surface hydrophobicity of the CG was 31.98 μg, and EG3 was similar to that of tallow; when the proportion of fat substituted by the emulsion gel reached 50%, the surface hydrophobicity of the beef mince attained a value of 37.34 μg. The emulsifying properties of proteins could be improved by appropriately changing the surface hydrophobicity of proteins, because the exposure of hydrophobic groups in proteins increased the interaction between proteins and oils, promoted the adsorption of proteins by oils, and formed dense and stable protein membranes. This is conducive to increasing the emulsifying properties [29,30,31]. However, the increase in surface hydrophobicity will lead to a decrease in protein stability and weakened protein–water interaction. The protein is prone to hydrophobic aggregation and protein denaturation, which is not conducive to the formation of a gel network structure [32,33].

#### 3.1.2. Effect of Chinese-Yam-Based Emulsion Gel on the Emulsion Stability of the Minced Beef

**Figure 2:**
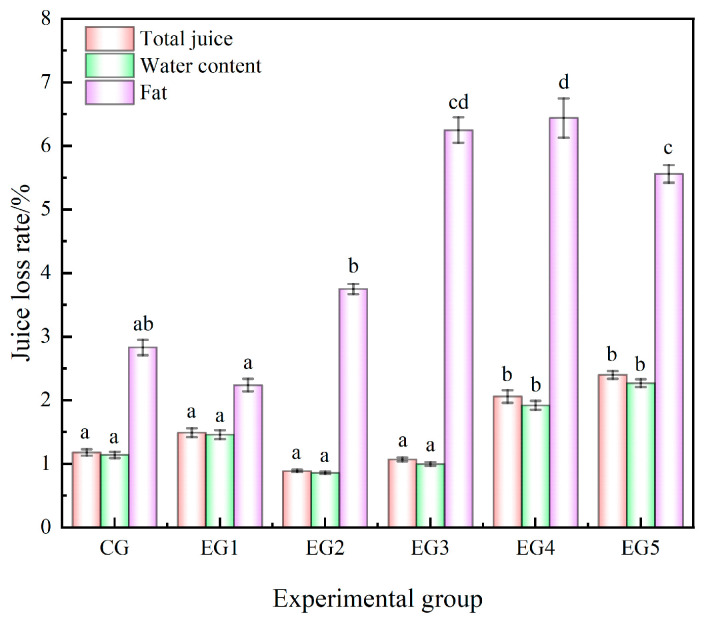
Effect of adding different amounts of emulsion gel on juice loss of beef mince. Note: Different letters indicate that there are significant differences between experimental groups (*p* < 0.05).

The stability of the emulsions is contingent upon the capacity of minced meat to effectively bind and retain moisture, serving as a pivotal indicator reflective of the quality of processed minced meat products. Figure 2 illustrates the juice loss rate exhibited by various emulsified minced meat samples.

The results indicate that the total juice loss rate and water loss observed in the CG, EG1, EG2, and EG3 were relatively minimal Notably, the total juice loss rate of the CG was 1.18% and the water loss rate of the CG was 1.14%. The total juice loss rate of EG2 was 0.89% and the water loss rate of EG2 was 0.86%, which is significantly different from EG4 and EG5. Regarding fat loss, EG1 demonstrated the smallest value of 2.24%, which was significantly lower than that recorded in EG2, EG3, EG4, and EG5. This phenomenon could be attributed to the fact that the incorporation of polysaccharides enhances the stability of the emulsion. However, with the increasing concentration of emulsion gel, it may have a shielding effect on the proteins in the entire mince system, thus reducing its ability to hold water and oil. Research endeavors have demonstrated that the stability of emulsions in emulsified minced meat can be augmented to a certain extent through the application of heat treatments. The findings of this experimental trial align with these previous research outcomes [34,35].

#### 3.1.3. Effect of Chinese-Yam-Based Emulsion Gel on Water Retention and Cooking Loss of Minced Beef

Water retention pertains to the capacity of meat products to maintain their inherent moisture or to absorb additional water when subjected to external forces such as extrusion, heating, and stirring. This phenomenon reflected the efficacy of the gel matrix in retaining water within its structure. The water retention capabilities of meat directly influenced the sensory attributes of the final product, and a higher yield of meat typically correlated with superior water-holding capacity [36,37].

**Figure 3:**
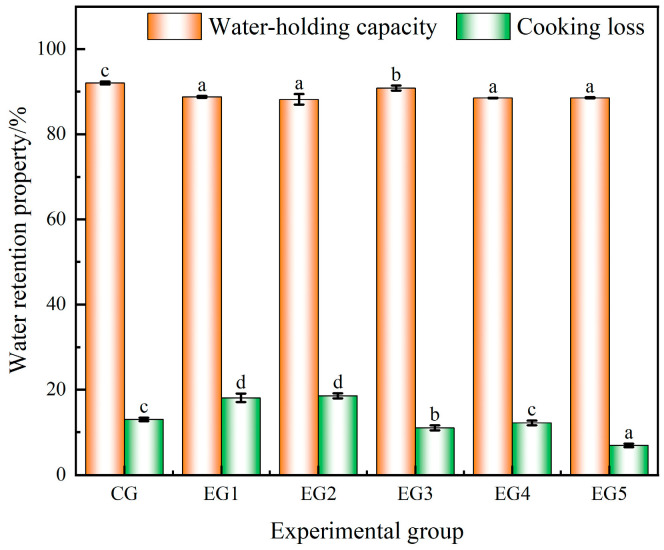
Water retention and cooking loss of beef minced with different amounts of emulsion gel. Note: Different letters indicate that there are significant differences between experimental groups (*p* < 0.05).

As depicted in Figure 3, the water-holding capacity of the control group (CG) exhibited significantly superior performance (*p* < 0.05) compared to the sample where emulsion gel served as the fat substitute. Furthermore, EG3 demonstrated a notably higher water-holding capacity (*p* < 0.05) than the other experimental groups. This outcome was attributed to the fact that an excessive amount of emulsion gel merely fills the gel system of the minced meat, ultimately leading to a reduction in the water-holding capacity of the sausage [38]. From the standpoint of cooking loss, as the quantity of emulsion gel added gradually increased, the cooking loss exhibited an overall decreasing trend. The cooking loss in the CG was significantly lower than in EG1 and EG2 (*p* < 0.05), while it was significantly higher than in EG3 and EG5 (*p* < 0.05). There was no significant difference between the CG and EG4 (*p* > 0.05). Overall, EG3 exhibited the optimal water retention capacity, which could be attributed to reduced surface hydrophobicity and subsequent enhanced protein–lipid interactions [39].

#### 3.1.4. Effect of Chinese-Yam-Based Emulsion Gel on Solubility of Minced Beef Protein

**Figure 4:**
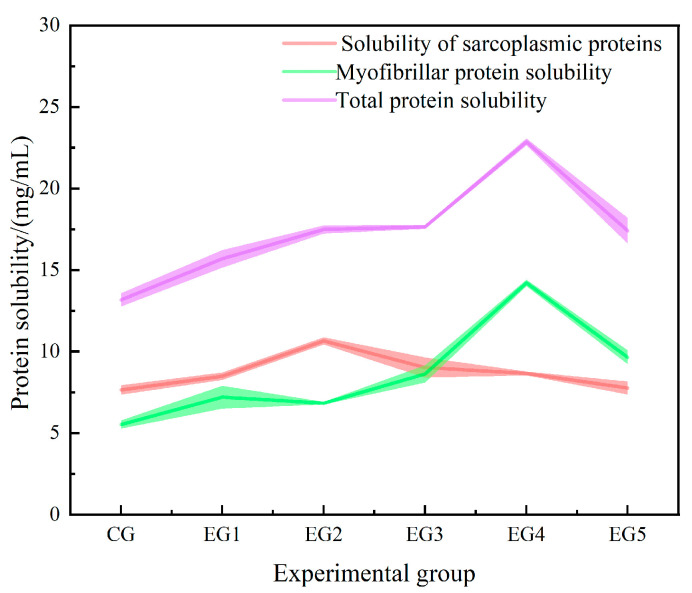
Solubility of minced beef protein with addition of different amounts of emulsion gel.

Solubility is mediated by non-covalent interactions (e.g., electrostatic forces, hydrophobic interactions, hydrogen bonds) and is considered the major characteristic of proteins that are selected for use in food [40]. As depicted in Figure 4, the progressive augmentation of emulsion gel addition exhibits a biphasic effect on overall protein solubility, initially eliciting an increase, followed by a decline. When the replacement of fat with emulsion gel was increased to 75%, the protein solubility reached the highest point of 22.85 mg/mL. Notably, the myofibrillar protein solubility attained its peak value of 14.20 mg/mL in EG4. This phenomenon might be due to the fact that, with the increase in the gel content of the emulsion, the salt ion content increases, reducing the repulsion force between protein molecules by harnessing the full potential of surfactants; oil droplets were more effectively adsorbed onto their surfaces, leading to an improvement in emulsification efficiency. In EG5, a slight decline in protein solubility was observed. This reduction can be ascribed to the protein condensation that occurred when the ionic strength increased beyond a critical threshold, ultimately resulting in decreased solubility. Several studies have revealed that an augmentation in the quantity of salt added results in an elevation in the salt-soluble protein content within the meat paste. Specifically, a significant amount of myosin or actin becomes adsorbed onto the surface of fat particles, forming a dense protein layer that subsequently enhances the emulsion stability of the sample [41]. In conclusion, the protein solubility of the beef meat paste system achieves its maximum level, and the emulsion stability is optimized, when the proportion of fat substituted by emulsion gel reaches 75%. The emulsifying properties of beef mince could be improved to some extent by using the Chinese-yam-based emulsion gel as a fat substitute.

### 3.2. Microstructure

#### 3.2.1. Effect of Chinese-Yam-Based Emulsion Gel on the Secondary Structure of Minced Beef Protein

**Figure 5:**
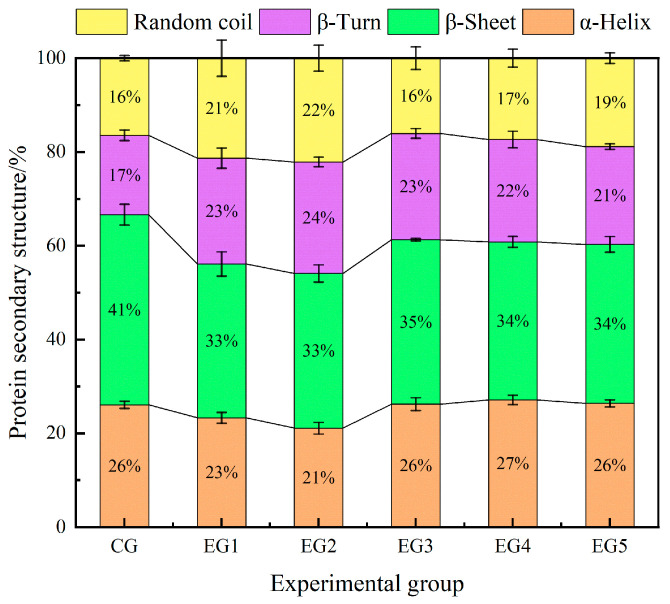
Secondary structure analysis of minced beef protein with different amounts of emulsion gel.

In Figure 5, the proportions of α-helix, β-sheet, β-turn, and random coil are displayed. The relative abundance of α-helix and β-sheet configurations constitute the secondary structural features of a well-ordered and stable protein. Figure 5 shows that with the increase in emulsion gel addition, the α-helix content first decreases and then increases. Specifically, the α-helix contents of EG1 and EG2 were lower than those of the other groups. Within the context of protein architecture, the α-helix is recognized as the most robust spiral conformation, primarily stabilized by hydrogen bonding interactions. Concurrently, β-sheet and β-turn are also related to hydrogen bonding. As the proportion of emulsion gel increased, there was a concomitant elevation in both protein and polysaccharide quantities. Notably, an increase in β-sheet and β-turn structures implies the formation of hydrogen bonds between the proteins and anionic polysaccharides [42]. Furthermore, the augmentation in the α-helix content suggests that the incorporation of emulsion gel served to transition the meat mince system from a disordered to an ordered state.

The relative abundance of β-turn and random coil configurations serves as an indicator of structural disorder. Notably, the β-turn content decreased with an increase in emulsion gel incorporation (*p* > 0.05). However, it remained significantly elevated compared to that of the CG (*p* < 0.05). This phenomenon could be attributed to the presence of salt coagulants within the emulsion gel. Specifically, metal ions such as Ca^2+^ or Mg^2+^, as well as H^+^, have the capacity to neutralize the surface charge of proteins, thereby altering the electrostatic interactions between protein molecules. Consequently, these alterations contribute to modifications in the secondary structural configuration of the proteins [43]. Additionally, an elevation in the concentration of the salt coagulants tends to promote instability within the beef minced meat system. Specifically, EG3 exhibited the lowest content of irregular curl, amounting to 16.05%, and there was no significant difference between EG3 and the CG (*p* > 0.05). It was suggested that the beef mince system in EG3 maintained a relatively stable state, which was optimal under these conditions. In conclusion, the secondary structural configuration of EG3 was found to be in the most favorable condition.

#### 3.2.2. Effect of Chinese-Yam-Based Emulsion Gel on Intermolecular Forces in Minced Beef

During the formation of minced beef gel, protein denaturation and polymerization give rise to a complex spatial three-dimensional network structure. This process involves the orderly denaturation and aggregation of proteins, which organizes into a three-dimensional network stabilized by various chemical forces, including ionic bonds, hydrogen bonds, hydrophobic interactions, and disulfide bonds. Notably, hydrophobic interactions and disulfide bonds play a pivotal role in maintaining the structural integrity of this network.

**Figure 6:**
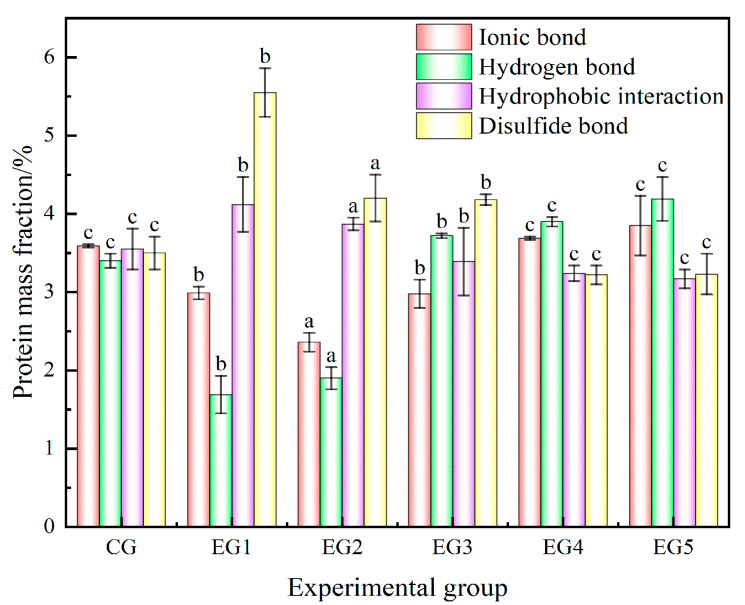
Intermolecular force of minced beef protein with addition of different amounts of emulsion gel. Note: Different letters indicate that there are significant differences between experimental groups (*p* < 0.05).

Ionic and hydrogen bonds constitute the primary natural structures that are instrumental in maintaining the stability of proteins [44]. During the formation of minced meat gels, the initial step involves the disruption of ionic bonds by salt ions, facilitating the dispersion of proteins. Subsequent heating then induces protein denaturation and aggregation, ultimately leading to gel formation. The analysis in Figure 6 shows that there is no significant difference in the ion bond content between the CG and EG4 and EG5 (*p* > 0.05). However, the ion bond content of the CG is significantly higher than that of EG1, EG2, and EG3 (*p* < 0.05). So, the structures of the CG, EG4, and EG5 are relatively stable.

Hydrogen bonds mainly maintained the α-helix structure in the secondary structure of proteins, which were related to protein gelation and viscoelasticity, and were broken when proteins were denatured by heat, and were reformed after the meat gel was cooled, which played a role in stabilizing the binding of water and promoting the hardening of the gel [45]. As shown in the figure, the hydrogen bond content of the CG is significantly lower than that observed in EG4 and EG5 (*p* < 0.05). In contrast, the hydrogen bond content of the CG is significantly higher than that of EG1, EG2, and EG3 (*p* < 0.05).

The hydrophobic interaction primarily arose from the denaturation of proteins via heating, resulting in the exposure of hydrophobic groups. This exposure led to an enhancement of the interactions among the non-polar groups of adjacent molecules, ultimately culminating in their aggregation and the formation of a gel structure. In the unheated state, these hydrophobic groups were embedded within the α-helix structure of proteins. The aggregation of proteins was intimately associated with the formation of disulfide bonds. During heat-induced gel formation, heating serves as the primary catalyst for the formation of disulfide bonds through sulfhydryl oxidation. Notably, the optimal temperatures for disulfide bond formation vary across different types of minced meat [46]. Figure 6 demonstrates that the hydrophobic interaction and disulfide bond contents of the CG are not significantly different from those of EG4 and EG5 (*p* > 0.05), but are significantly lower than those of EG1, EG2, and EG3 (*p* < 0.05). An increase in the emulsion gel content elevated the levels of polysaccharides and phenolic compounds, thereby enhancing antioxidant properties. The balance between thiol groups and disulfide bonds is influenced by factors such as oxidation conditions, enzymatic hydrolysis, and pH values [47]. Future research will focus on optimizing the thiol/disulfide bond balance to improve the antioxidant performance of the meat emulsion system.

#### 3.2.3. Effect of Chinese-Yam-Based Emulsion Gel on the Microstructure of Minced Beef

The microstructure of the minced meat gel serves as a direct indicator of its gel state. By observing the internal structure of the sample under a scanning electron microscope, one can intuitively ascertain various characteristics such as the degree of compactness and porosity, the presence or absence of pore structures, the roughness or smoothness of the surface, the occurrence of gel fractures, and the emergence of lamellar structures. Furthermore, through direct reflection, one can analyze the underlying reasons for a range of various laws, including water retention capacity and gel strength [48,49].

**Figure 7:**
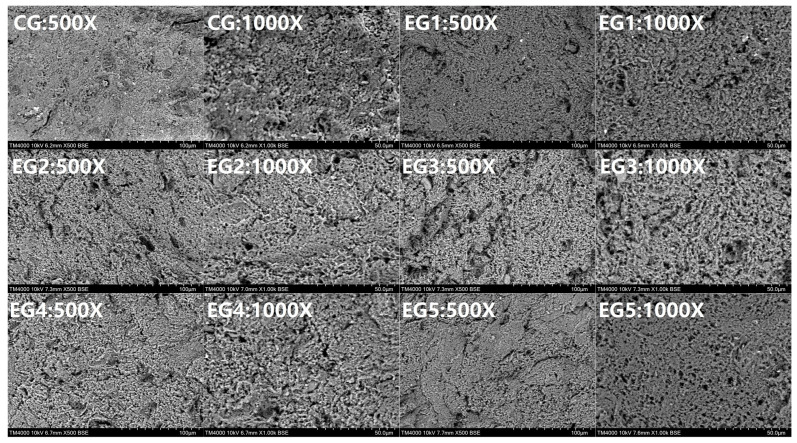
The microstructure of beef mince with addition of different amounts of emulsion gel.

As depicted in Figure 7, the incremental addition of emulsion gel exhibits a notable influence on the microstructural alterations of beef mince. Specifically, the gel formed within the CG exhibits a coarse structure and uneven texture, whereas the gel in EG1 displays a relatively dense consistency, resulting in the formation of small pores. EG2, EG3, EG4, and EG5 differ in the amount of emulsion gel added. It is evident that as the amount of emulsion gel increases, the gel structure becomes progressively more compact, with pores that are relatively small, regular, and uniformly distributed. This phenomenon could be attributed to the non-flowable nature and high viscosity of the emulsion gel, which, upon grinding, facilitates its effective dispersion within the meat paste system, thereby enhancing the compactness and uniformity of the gel structure [50]. In comparison to the CG, the incorporation of emulsion gels significantly refines the gel structure of beef mince, rendering it more regular and compact.

### 3.3. Discussion

This experimental study investigated the impact of an emulsion gel, formulated primarily with Chinese yam as the key ingredient, on the emulsification properties and microstructural characteristics of beef mince. The findings indicate that, when the proportion of fat substituted by the emulsion gel reaches 50%, the surface hydrophobicity of the beef mince attains a value of 37.34%, which approximates that of beef tallow. Furthermore, the water retention capacity of this formulation is significantly superior to that of the other test groups (*p* < 0.05). Additionally, when the fat replacement with emulsion gel is increased to 75%, the protein solubility is observed to be 22.85 mg/mL. As the quantity of emulsion gel increases, the gel structure of the beef mince undergoes a gradual densification. This alteration is accompanied by a statistically significant upward trend in the overall α-helix content (*p* < 0.05), but it only holds true after reaching a certain level. Meanwhile, the β-turn angle exhibits the opposite trend, and the β-sheet content demonstrates a biphasic pattern, initially increasing and subsequently decreasing. Notably, the random coil content of the emulsion gel reaches its minimum when the fat content is at 50%, corresponding to a state of maximal stability in the beef mince. However, according to the results described in Section 3.1.2, while the loss of water and fat is similar to the loss of juice, the bars for juice loss are much lower than those for fat loss. On this basis, we will continue to study the emulsification stability of ground beef at different temperatures and further explore its emulsification characteristics at different temperature levels. In addition, since the amount of cold-pressed flaxseed oil was set at a fixed value in this study, the influence of adding different contents of cold-pressed flaxseed oil on the emulsion system will be further explored in the next study.

## 4. Conclusions

This experimental study investigated the impact of an emulsion gel, formulated primarily with Chinese yam as the key ingredient, on the emulsification properties and microstructural characteristics of beef mince. Each group of tests was repeated several times, and the average value was the final test value. The findings indicate that, when the proportion of fat substituted by the emulsion gel reaches 50%, the surface hydrophobicity of the beef mince attains a value of 37.34%, which approximates that of beef tallow. Furthermore, the water retention capacity of this formulation is significantly superior to that of the other test groups (*p* < 0.05). Additionally, when the fat replacement with emulsion gel is increased to 75%, the protein solubility is observed to be 22.85 mg/mL. As the quantity of emulsion gel increases, the gel structure of the beef mince undergoes a gradual densification. This alteration is accompanied by a statistically significant upward trend in the overall α-helix content (*p* < 0.05), whereas the β-turn angle exhibits the opposite trend, and the β-sheet content demonstrates a biphasic pattern, initially increasing and subsequently decreasing. Notably, the random coil content of the emulsion gel reaches its minimum when the fat content is at 50%, corresponding to a state of maximal stability in the beef mince.

## Figures and Tables

**Table 1:** Preparation ratio of meat emulsion system.

Type	CG/g	EG1/g	EG2/g	EG3/g	EG4/g	EG5/g
Lean meat	62	62	62	62	62	62
Fat	20 (Beef fat)	20 (Flax seed oil)	15 (Flax seed oil)	10 (Flax seed oil)	5 (Flax seed oil)	0 (Flax seed oil)
Emulsion gel	0	0	5	10	15	20
Water	16	16	16	16	16	16
NaCl	2	2	2	2	2	2

## Data Availability

The original contributions presented in the study are included in the article; further inquiries can be directed to the corresponding author.

## References

[1] González N., Marquès M., Nadal M., Domingo J.L. (2020). Meat consumption: Which are the current global risks? A review of recent (2010–2020) evidences. Food Res. Int..

[2] Kim S.-A., Shin S. (2021). Red meat and processed meat consumption and the risk of dyslipidemia in Korean adults: A prospective cohort study based on the Health Examinees (HEXA) study. Nutr. Metab. Cardiovasc. Dis..

[3] Ren Y.Q., Huang L., Zhang Y.X., Li H., Zhao D., Cao J.N., Liu X.Q. (2022). Application of emulsion gels as fat substitutes in meat products. Foods.

[4] Zhang J., Hayden K., Jackson R., Schutte R. (2021). Association of red and processed meat consumption with cardiovascular morbidity and mortality in participants with and without obesity: A prospective cohort study. Clin. Nutr..

[5] Domínguez R., Munekata P.E., Pateiro M., López-Fernández O., Lorenzo J.M. (2021). Immobilization of oils using hydrogels as strategy to replace animal fats and improve the healthiness of meat products. Curr. Opin. Food Sci..

[6] Feng Y.Y., Xu J.X., Yu D., Kong B.H., Liu Q. (2019). Recent Advances in the Application of Emulsion Gels as Fat Replacers in Meat Products. Food Sci..

[7] Wei L., Ren Y., Huang L., Ye X., Li H., Li J., Cao J., Liu X. (2024). Quality, Thermo-Rheology, and Microstructure Characteristics of Cubic Fat Substituted Pork Patties with Composite Emulsion Gel Composed of Konjac Glucomannan and Soy Protein Isolate. Gels.

[8] Khosravani M., Nazari S.M., Asadi G. (2024). Evaluation of Chemical and Sensory Properties of Low-fat German Sausages Produced by Maltodextrin. Iran. Food Sci. Technol. Res. J..

[9] Dreher J., Weißmüller M., Herrmann K., Terjung N., Gibis M., Weiss J. (2021). Influence of protein and solid fat content on mechanical properties and comminution behavior of structured plant-based lipids. Food Res. Int..

[10] Martins A.J., Vicente A.A., Cunha R.L., Cerqueira M.A. (2018). Edible oleogels: An opportunity for fat replacement in foods. Food Funct..

[11] Wang W., Yan L., Yi S. (2024). Fucoidan–Vegetable Oil Emulsion Applied to Myosin of Silver Carp: Effect on Protein Conformation and Heat-Induced Gel Properties. Foods.

[12] Luan H.L., Wu Y.Q., Zheng H.X., Ke X.Y., Mao L.K. (2021). Preparation and Physicochemical Characterization of Cinnamic Acid Based Oleogels and Oleogel Emulsions. Food Sci..

[13] Wang W.N., Wang Y., Yu Y., Jiang Y.T., Wu F., Han C.P., Yu D.Y., Shi Y.G. (2021). Preparation and Properties of Rice Oil-based Oleogel with Sugarcane Wax. Food Sci..

[14] Guo J., Cui L., Meng Z. (2023). Oleogels/emulsion gels as novel saturated fat replacers in meat products: A review. Food Hydrocoll..

[15] Duan X., Li G.G., Li L.L., Ren G.Y., Zhu L.W., Wei X.Y. (2024). Multiphase microwave drying and quality characteristics of Chinese yam based on conversion point regulation. Trans. Chin. Soc. Agric. Eng..

[16] Liu X.-X., Yan Y.-Y., Liu H.-M., Wang X.-D., Qin G.-Y. (2019). Emulsifying and structural properties of polysaccharides extracted from Chinese yam by an enzyme-assisted method. LWT.

[17] Luo L., Qin T., Huang Y., Zheng S., Bo R., Liu Z., Xing J., Hu Y., Liu J., Wang D. (2017). Exploring the immunopotentiation of Chinese yam polysaccharide poly (lactic-co-glycolic acid) nanoparticles in an ovalbumin vaccine formulation in vivo. Drug Deliv..

[18] Pintado T., Herrero A.M., Jiménez-Colmenero F., Cavalheiro C.P., Ruiz-Capillas C. (2018). Chia and oat emulsion gels as new animal fat replacers and healthy bioactive sources in fresh sausage formulation. Meat Sci..

[19] Zhou F. (2017). Effect of Lipid Types and Fatty Acid Saturation Degree on the Pork Meat Proteins Emulsifying Properties. Master’s Thesis.

[20] Zhang M., Haili N., Chen Q., Xia X., Kong B. (2018). Influence of ultrasound-assisted immersion freezing on the freezing rate and quality of porcine longissimus muscles. Meat Sci..

[21] Xu Y.N., Cao C.A., He J.J., Kong B.H., Sun W.D., Liu Q. (2024). Research Progress in the Regulatory Mechanism of Hydrocolloids on the Formation of Heat-Induced Myofibrillar Protein Gel. Food Sci..

[22] Zhang L., Shi C., Xiao K., Li C., Mo J., Zhang Z., Shi Y. (2019). Improvement and Application of Biuret Method for Determination of Collagen Peptide from Tilapia. Food Sci..

[23] Wang Q., Tang Y., Yang Y., Lei L., Lei X., Zhao J., Zhang Y., Li L., Wang Q., Ming J. (2022). The interaction mechanisms, and structural changes of the interaction between zein and ferulic acid under different pH conditions. Food Hydrocoll..

[24] Zhang J.L. (2017). Study on Hydration Characteristics of Meat Battersduring Heat-Induced Processing. Master’s Thesis.

[25] Yang P., Xu C.H., Xu X., Wang L.J., Zhu W., Li J.M., Meng X.J., Xu Q. (2023). Effect of Extreme Acid Combined with Heat Induction on Structureand Properties of Soybean Protein Isolate Microgel. Food Sci..

[26] Ai M., Zhou Q., Guo S., Ling Z., Zhou L., Fan H., Cao Y., Jiang A. (2019). Effects of tea polyphenol and Ca(OH)2 on the intermolecular forces and mechanical, rheological, and microstructural characteristics of duck egg white gel. Food Hydrocoll..

[27] Kang H.B., Zou L.L., Zhang H.Y., Cai C.Q., Wang B., Ke H.R. (2018). Effect of High Temperature Treatment on Chemical Forces of Beef Proteins and Structure of Myofibrillar Protein. Food Sci..

[28] Yang Y.L., Zhou L., You Y., Tang X.Z., Wei S.M. (2018). The Effects of Oxidation onTextural Properties and Water Holding Capacity of Heat-Induced Myofibrillar Protein Gel. Sci. Agric. Sin..

[29] Kallakas H., Plaza N., Crooks C., Turner D., Gargulak M., Arvanitis M.A., Frihart C.R., Hunt C.G. (2024). Effect of Protein Surface Hydrophobicity and Surface Amines on Soy Adhesive Strength. Polymers.

[30] Wätjen A.P. (2023). Exploring Diversity of Lactic Acid Bacteria for the Development of Plant-Based Dairy Alternatives. Ph.D. Thesis.

[31] Zahir M., Fogliano V., Capuano E. (2021). Soybean germination limits the role of cell wall integrity in controlling protein physicochemical changes during cooking and improves protein digestibility. Food Res. Int..

[32] Cao Y.G., Xiong Y.L. (2015). Chlorogenic acid-mediated gel formation of oxidatively stressed myofibrillar protein. Food Chem..

[33] Jia N., Lin S.W., Wang L.T., Liu D.Y. (2020). Effects of Changes in Sulfhydry Content and Surface Hydrophobicity of Myofibrillar Protein Induced by Gallic Acid on Its Gel Properties. Food Sci..

[34] Gao T., Wu X., Gao Y., Teng F., Li Y. (2024). Construction of emulsion gel based on the interaction of anionic polysaccharide and soy protein isolate: Focusing on structural, emulsification and functional properties. Food Chem. X.

[35] Lopes-da-Silva J.A., Monteiro S.R. (2019). Gelling and emulsifying properties of soy protein hydrolysates in the presence of a neutral polysaccharide. Food Chem..

[36] Herrero A.M., Carmona P., Jiménez-Colmenero F., Ruiz-Capillas C. (2014). Polysaccharide gels as oil bulking agents: Technological and structural properties. Food Hydrocoll..

[37] Zhou S.Q., Liu X., Liu S.W., Lv X.Z., Liu J., Zhou D.P., Tang X.Z. (2020). Effect of Double Emulsion Gels as Fat Replacers on the Physicochemical Properties of Chicken Sausage. Sci. Technol. Food Ind..

[38] Chen Y.C., Jiang S., Cao C.A., Chen J.X., Kong B.H., Liu Q. (2019). Evaluation of the Quality of Frankfurters Prepared with Highly Stable Vegetable Oil-in-Water Pre-Emulsion as a Partial Replacer of Pork Back Fat. Food Sci..

[39] Zhang L., Li Q., Zhang W., Bakalis S., Luo Y., Lametsch R. (2024). Different source of commercial soy protein isolates: Structural, compositional, and physicochemical characteristics in relation to protein functionalities. Food Chem..

[40] Aryee A., Agyei D., Udenigwe C. (2018). Impact of processing on the chemistry and functionality of food proteins. Proteins in Food Processing.

[41] Whiting R. (1984). Stability and gel strength of frankfurter batters made with reduced NaCl. J. Food Sci..

[42] Li Q., Wang Z., Dai C., Wang Y., Chen W., Ju X., Yuan J., He R. (2019). Physical stability and microstructure of rapeseed protein isolate/gum Arabic stabilized emulsions at alkaline pH. Food Hydrocoll..

[43] Dai Y.Q., Liu X.L., Wu H., Yin L.Q., Zhou J.Z., Dong M.S., Xia X.D. (2021). Effects of Different Coagulants on Intermolecular Forces and Secondary Structure of Soybean Protein Isolate. Sci. Technol. Food Ind..

[44] Liu F.F., Lin W.L., Li L.H., Wu Y.Y., Yang S.L., Huang H., Yang X.Q., Lin Z. (2020). Mechanism Underlying Protein Changes during Processing and Gelation of Sea Bass Surimi. Food Sci..

[45] Sun X.D., Arntfield S.D. (2012). Molecular forces involved in heat-induced pea protein gelation: Effects of various reagents on the rheological properties of salt-extracted pea protein gels. Food Hydrocoll..

[46] Brenner T., Johannsson R., Nicolai T. (2009). Characterization of fish myosin aggregates using static and dynamic light scattering. Food Hydrocoll..

[47] Ju Q. (2023). Study on the Structure and Functional Characteristics of Soybean 7S Globulin Subunit. Ph.D. Thesis.

[48] Park J., Rhee K., Keeton J., Rhee K. (1989). Properties of low-fat frankfurters containing monounsaturated and omega-3 polyunsaturated oils. J. Food Sci..

[49] Shao J.H., Wu J.Q., Zhou G.H., Wei C.G., Xu X.L., Liu D.Y., Song L., Jia N. (2013). Effects of Sulfhydryl Content and Hydrophobicity on Gel and Emulsifying Properties of Pork Proteins. Food Sci..

[50] Spotti M.J., Tarhan Ö., Schaffter S., Corvalan C., Campanella O.H. (2017). Whey protein gelation induced by enzymatic hydrolysis and heat treatment: Comparison of creep and recovery behavior. Food Hydrocoll..

